# Automated Instability Detection for Interactive Myocontrol of Prosthetic Hands

**DOI:** 10.3389/fnbot.2019.00068

**Published:** 2019-08-27

**Authors:** Roberto Meattini, Markus Nowak, Claudio Melchiorri, Claudio Castellini

**Affiliations:** ^1^Department of Electrical, Electronic and Information Engineering (DEI), University of Bologna, Bologna, Italy; ^2^German Aerospace Center (DLR), Institute of Robotics and Mechatronics, Oberpfaffenhofen, Germany

**Keywords:** myocontrol, instability, reliability, interactive myocontrol, prosthesis control, amputee

## Abstract

Myocontrol is control of a prosthetic device using data obtained from (residual) muscle activity. In most myocontrol prosthetic systems, such biological data also denote the subject's intent: reliably interpreting what the user wants to do, exactly and only when she wants, is paramount to avoid instability, which can potentially lead to accidents, humiliation and trauma. Indeed, instability manifests itself as a *failure* of the myocontrol in interpreting the subject's intent, and the automated detection of such failures can be a specific step to improve myocontrol of prostheses—e.g., enabling the possibility of self-adaptation of the system via on-demand model updates for incremental learning, i.e., the interactive myocontrol paradigm. In this work we engaged six expert myocontrol users (five able-bodied subjects and one trans-radial amputee) in a simple, clear grasp-carry-release task, in which the subject's intent was reasonably determined by the task itself. We then manually ascertained when the intent would not coincide with the behavior of the prosthetic device, i.e., we labeled the failures of the myocontrol system. Lastly, we trained and tested a classifier to automatically detect such failures. Our results show that a standard classifier is able to detect myocontrol failures with a mean balanced error rate of 18.86% over all subjects. If confirmed in the large, this approach could pave the way to self-detection and correction of myocontrol errors, a tighter man-machine co-adaptation, and in the end the improvement of the reliability of myocontrol.

## 1. Introduction

Myocontrol—open-loop high-level control based upon muscle activity—is the primary way to allow upper-limb amputees to control a self-powered prosthesis (Jiang et al., 2012), at least in the academic community. Such control is usually enforced using (residual) muscle activity of the user's body, gathered via surface electromyography (sEMG, Merletti et al., 2011), or more advanced techniques (Castellini et al., 2014). It is intended and desired, that coordinated muscle activation patterns correspond to desired actions of the rehabilitation device; a suitable system must then be put in place to correctly interpret such patterns, *exactly for the duration of an action*—this is the essence of *reliability* in myocontrol.

Instability in myocontrol is here outlined as the manifested consequence of low robustness of the human-machine interface (HMI) control system, with respect to changes in the sEMG input signals (for a same given users intent), producing control outputs inconsistent with respect to the user's will. Consequently, we define a myocontrol *failure* as an event in which the prosthetic hand starts a behavior that does not coincide with the one expected by the user, i.e., it is in contrast with the users intent (note that in the following of the manuscript the terms myocontrol instability and myocontrol failure can be used interchangeably). In the extreme case a failure can be catastrophic: picture for instance a prosthetic hand suddenly failing to turn the steering wheel of a car when required.

Still, after 30 years and more of academic research, reliability of myocontrol is an open issue. Indeed, myocontrol suffers from the quintessential problems related to human-machine interaction: the inconstancy of signals gathered from human beings; the need to determine what the user wants the device to do (at best, a blurry target); the fact that, by definition, assistive/rehabilitation devices are to be controlled by disabled and impaired persons such as e.g., stroke patients, amputees, elderly subjects, etc., whose signals are, from the point of view of the engineer, even worse.

A spectacular example of the general unreliability of myocontrol can be found in the outcome of the ARM competition of the 2016 Cybathlon (ETH, 2016). Instability led to so many failures by users of myocontrolled arm/hand prosthetic systems that both categories were won by teams using body-powered one-DoF prosthetic arms, and they were competing against some of the most advanced academic solutions in the world^1^. A very recent, fascinating survey about current pitfalls and practical requirements of myocontrol is Schweitzer et al. (2018); see also, for instance, ETH (2016) and the video clips in Schweitzer (2016).

The reasons why the scientific community has so far been unable to provide a safe solution to this problem lie both in the unstable nature of the above-mentioned signals (Micera et al., 2010; Peerdeman et al., 2011; Fougner et al., 2012; Ison and Artemiadis, 2014) and we claim (Castellini et al., 2015; Nowak et al., 2017a, 2018), in the bad design of testing protocols, and the lack of an appropriate framework to induce co-adaptation in the user. Interestingly, these remarks obviously also apply to “standard” human-machine interaction, e.g., teleoperation. Our way toward the solution of the problem is incremental learning, allowing for on-demand model updates in real time, leading to an *interactive myocontrol* paradigm: a natural, simultaneous and proportional (s/p) control scheme which can be taught new information (Gijsberts et al., 2014; Strazzulla et al., 2017), and where the possibility of updating should desirably be achieved in an autonomous and real-time fashion. Therefore, an “automatic *oracle*” able to detect myocontrol instability is the first step toward such an approach, that otherwise would require the presence of the subjective judgement of a “human oracle” (i.e., the experimenter or the subject), introducing a significant weakness in the whole paradigm (Nowak et al., 2018). Following up our own preliminary work (Nowak et al., 2017b), we hereby propose a further advancement toward the automated detection of failures in myocontrol, especially by involving multiple subjects in a more appropriate experimental protocol and using a completely new feature extraction and labeling approach for the classification along with an extended analysis and discussion. Other past studies also attempted fault-tolerant approaches to myocontrol (e.g., Hargrove et al., 2010; Scheme et al., 2011; Amsuss et al., 2014); however, they were concerned with classification of myoelectric patterns, therefore not considering s/p myocontrol as it has been made in the present work.

In order to minimize the complexity of the problem, we made the assumption that if we engage a human subject in a simple, well-defined task with a clear aim, the subject's intent will adhere to the actions required by the task. If we can time the intervals during which a specific action is required, we can then claim that the subject's intent *is* the sequence of actions scripted in the task. Accordingly, we designed an extremely simplified, well-structured but still realistic grasp-carry-release experimental task, to evaluate automatic detection of the myocontrol instability. Namely, we engaged six subjects, five able-bodied persons and one trans-radial amputee. All subjects were expert users of the state-of-the-art s/p myocontrol system used in this study (see section 2.1). By means of *a posteriori* analysis of video recordings of the experiment, we were able to obtain the starting and ending times of the grasp actions and to determine the myocontrol failure occurrence instants, i.e., when the hand would grasp during the no-grasp intervals and vice-versa (false positives and false negatives). Additionally, each user had a wireless button available to signal his/her feeling on when the myocontrol system would be failing. Using these pieces of information, we could exactly label the task execution. Lastly, a standard classifier was used to try and associate features—extracted from the myocontrol-predicted motor currents and the status feedback signals from the prosthetic hands—to failures (both false positives and negatives).

The article is organized as follows: in section 2 the experimental setup and protocol, and the classification system are presented, whereas section 3 reports per-subject and global analyses results, and finally section 4 is dedicated to the discussion of several emerging aspects and conclusions.

## 2. Methods

**Figure 1:**
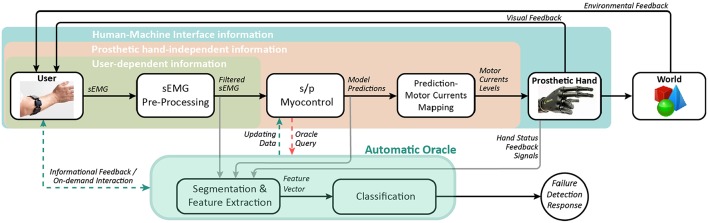
Conceptual block diagram of the automatic oracle applied to the HMI for the control of a prosthetic hand. The present study focuses on the possibility of detecting myocontrol failures extracting features from the *Prosthetic hand-independent* and *Human-Machine Interface* informational zones. Dashed lines indicate desirable information flows for the future on-line implementation of the oracle.

Figure 1 depicts a conceptual representation of the HMI organization and informational flow related to the setup used in this work. The idea of an automatic oracle for myocontrol failure detection is also outlined. In this relation, the oracle acts as a *supervising agent* having access to all information available within the HMI system for a hand prosthesis. The oracle should be able to gather significant data, manipulate and interpret it and, finally, provide a response on the occurrence of a myocontrol failure (as output of a classification system) whenever consulted. This behavior should be available online during the control of the prosthesis, also providing informational feedback to the user and/or demanding for an interaction if useful, and supplying specific buffered data to allow the s/p myocontrol update its model in the face of new detected instabilities, carrying out an interactive learning paradigm. Relying on such architecture, in this work we focussed on evaluating the possibility to detect myocontrol failures by extracting features that are based on the myocontroller prediction outputs (belonging to the *prosthetic-hand independent* informational zone, see Figure 1) and on a minimal set of the prosthetic hand feedback signals (belonging to the *human-machine interface* informational zone, see Figure 1).

### 2.1. Experimental Setup and Control

**Figure 2:**
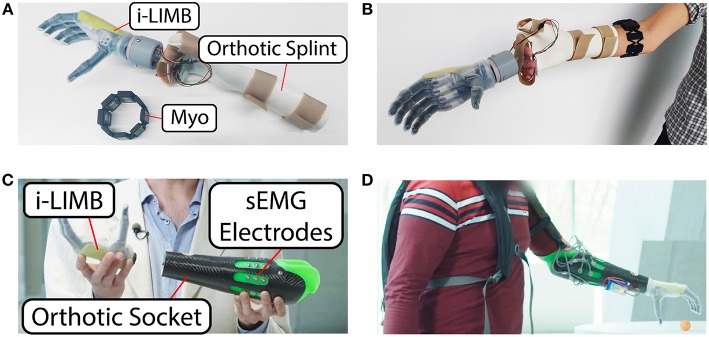
Overview of the experimental setup. **(A)** Setup view for the able-bodied subjects. **(B)** The setup worn by one of the subjects. Electronics and battery supply are embedded in the palm area of the splint. **(C)** Setup view for the amputee subject. **(D)** The setup worn by the amputee subject. Electronics and battery supply are embedded in a small backpack.

The experimental setup for the intact subjects as well as for the amputated subject is visible in Figure 2. For the intact subject, it consisted of a commercial orthotic splint that was fitted with a custom-design mounting for the *i-LIMB Revolution* multi-fingered prosthetic hand manufactured by Touch Bionics / Össur (Touchbionics, 2017) (in the academic variant called *Robo-limb*) and a single *Myo* bracelet by Thalmic Labs (Thalmic-Labs, 2017). The i-LIMB is a commercially available electric-powered prosthetic hand presenting 6-DoF, including the flexion of 5 motorized fingers plus the abduction of the thumb. The Myo has eight sEMG sensors covering the full circumference of the users proximal forearm, and allows to acquire sEMG data at a sampling rate of 200 Hz. The Robo-limb has six step motors, each one of which can directly be controlled in current via a simple serial port protocol. This prosthetic hand provides, for each motor, feedback signals about the current reading and a “digit status” flag; in particular, this digit status feedback consists of a signal with discrete values, each of them identifying univocally one of the following digit statuses: “opening,” “open,” “closing,” “closed,” and “stalled”. For the amputed subject, the setup consisted of a specifically designed orthotic socket, with the *Robo-limb* attached to its end-point by means of a standard connection interface. In particular, the prosthetic socket was a bespoke carbon fiber socket with 8 sEMG sensors embedded, and a standard pin/lock connection, manufactured by Pohlig GmbH (GmbH, 2019). The socket therefore allowed to acquire sEMG signals from the residual forearm's muscles. The experimental setups were completely wearable thanks to portable power supplies (lithium batteries) and wireless data communication with a nearby computer (bluetooth). All subjects were provided with a wireless button to signal whenever the myocontrol failed in interpreting their intent (more details in section 2.2).

S/p myocontrol was enforced using four parallel instances of Ridge Regression with Random Fourier Features (RR-RFF), a method already tested and used for myocontrol; the description of this particular Machine-Learning(ML)-based myocontroller lies outside of the article aims—for details refer to Gijsberts et al. (2014), Strazzulla et al. (2017), and Patel et al. (2017). Rectified sEMG data, pre-processed with a 1st-order Butterworth low-pass filter with cut-off at 1 Hz, was taken as the input space of the myocontroller. On the other hand, the output of the myocontroller was given by the outputs of the four RR-RFF instances, and it was directly fed as (proportionally scaled) current commands to the six motors of the prosthetic hand. Note that, in order to build the RR-RFF instances, the algorithm was trained by gathering sEMG data while the users performed specific actions in accordance with explicative visual stimuli administered by the i-LIMB itself: i.e., the user followed the actions showed by the robotic hand (i.e., the ground truth) while, automatically, the sEMG data was acquired in order to train the algorithm (for details see again Gijsberts et al., 2014; Patel et al., 2017, and refer to section 2.2 for information on the experimental procedure and specific actions). The reason why only four myocontrol prediction signals are used for the six DoFs of the prosthetic hand is that, for the grasp actions required in this study, the flexion of the middle, ring and little fingers are fully coupled (see section 2.2). Finally, it is worth to note that the output of the RR-RFF algorithm is a real number, which we limit to values between 0 and 1. In this relation, the mapping of this value to the prosthetic hand motor currents was based on a threshold (empirically set to 0.3) in order to let the users voluntarily trigger the prosthetic hand action. This means that the proportional control was between 0.3 and 1, whereas below 0.3 the robo-limb was set to a “home configuration” with all fingers fully open (see also the robo-limb actions description in section 2.2.3). Finally, please note that the use of the activation threshold and the “home configuration” are not arbitrary assumptions of the present study, but they were already included in the implementation of the RR-RFF-based s/p myocontrol developed and presented in our past works (Gijsberts et al., 2014; Patel et al., 2017; Strazzulla et al., 2017).

### 2.2. Experimental Protocol

#### 2.2.1. Participants

The experiment was performed in accordance with the Declaration of Helsinki and was approved by the Work Council of the German Aerospace Center. All participants were thoroughly informed about the experimental protocol and were asked to sign an informed consent form. The participants were five able-bodied subjects and one trans-radial amputee, in the age between 28 and 45 years old among which there were five men and one woman. All subjects were experts in the usage of the state-of-the-art s/p myocontrol used in this study. “Experts” means that they knew what myocontrol is and they already used the same setup of this work at least two times in the last 2 months. This was necessary since we wanted to detect failures rather than observing the quality of the control. Additionally, please note that the myocontroller used in this study allows incremental learning, therefore enabling continuous co-adaptation during the online prosthesis usage by triggering new model updates; this further justifies the engagement of expert users, since in this case the failure detection is very useful also when a certain learning (and co-adaptation) framework was already established.

#### 2.2.2. The Carrying Task

**Figure 3:**
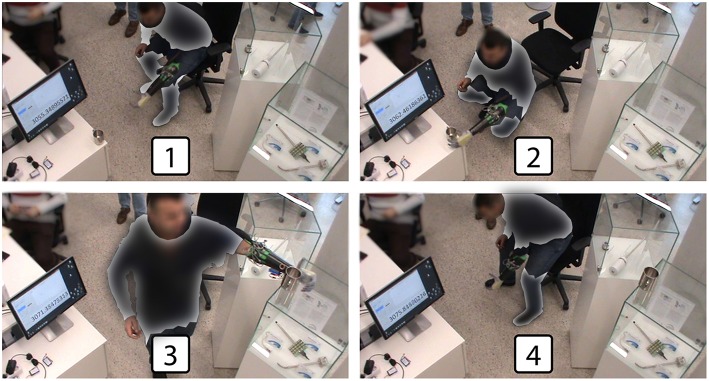
Experimental protocol overview: the subject **(1)** stood up and began the task, **(2)** grasped the cup in the lower position, **(3)** released the cup in the elevated position, and **(4)** sat down completing the task. Some parts of the image have been omitted in order to preserve the anonymity of the subject.

The central part of the experiment is composed by the repetitions of a specifically designed carrying task. In detail, a mug was supposed to be grasped at a low height using a power grasp (see also section 2.2.3), carried to a different location and released at an elevated height (Δ*d* ≈ 1*m*). For each task the subject started in a seated position, then stood up, performed the task and ended the repetition by sitting down again. After moving the mug to the elevated position the following iteration of the task was performed in the opposite direction, i.e., moving the mug from the elevated to the low position. This was repeated ten times, i.e., five tasks low to elevated position and five tasks elevated to low position. Note that the very simple structure of this task was desirable in order to hold our assumption that a human subject involved in a well-defined task with a clear aim will adhere to the actions required by the task. However, bisedes its simple structure, the specific design of this experimental task included walking, standing up, sitting down and body posture rise/lower—in addition to the main idea of object grasping/releasing—because we wanted to introduce mental effort/concentration and body posture demanding elements in the execution of the task, since they are known as stress factors for myocontrol instability (see section 2.2.5). In case a “catastrophic” failure prevented the completion of the task (i.e., the mug was dropped, impossibility to grasp or release the mug, etc.), the subject was asked to return to the seated position. The mug was returned to the original location and the attempted task was repeated. In this relation, please note that a failure during the transportation phase of the grasped object not necessarily coincided with a mug falling; as a single explicatory example, consider that just the index erroneously opened during the object transportation: this would not generate a “catastrophic” failure. After the ten task repetitions were completed, the subject took a break of no less than 10 min, and then executed a second session in the same way as the one just described. In Figure 3 we see an overview of the experimental protocol execution. Therefore, in total, two sessions per subject were performed, each of which was composed of a myocontroller training phase and the ten iterations of the mug-carrying task. In particular, during the training phase the user followed a stimulus while sEMG data were gathered to train the ML algorithm.

#### 2.2.3. Myocontroller Training and Update Procedure

As already mentioned, the mug is supposed to be grasped using a power grasp. However, the ML algorithm was trained with the “power grasp,” “index pointing,” “thumbs-up,” and “rest.” In this application, the “rest” action implicates the opening of the artificial hand, i.e., the artificial hand fully open is intended as a “home configuration” that was reached when the user didn't contract the forearm muscles (i.e., the “rest” action). Each of these actions was provided for the training only with one repetition demonstrated by the user. Note that this is done to make the carrying task “more tricky” from a myocontrol point of view. By adding more grasp types there is an higher probability of misinterpretation by the ML algorithm. The reason of this lies on the fact that, by training only on one repetition, the ML algorithm lacks information about signal variations along different muscle contractions and body postures. Indeed, the ML algorithm training was performed in a seated position with the elbow located on an arm rest, whereas the carrying tasks were performed in bent and stretched body positions (see Figure 3).

Once the training is completed, the user started the carrying task session. However, an update of the ML algorithm could be possible in case of a “catastrophic” failure, i.e., a failure that prevented the completion of the task. As mentioned before, after this happened the subject was asked to return to a seated position and retry the task. Here, if the subject “catastrophically” failed for a second time, the ML algorithm was updated. Since RR-RFF can be used incrementally, we added more information about the failed attempt to the data base of the algorithm. For example, after the second failed attempt on release the mug in the elevated position, another repetition of the “rest” action would be trained to improve the control in this specific situation. Among all sessions, there were thirteen repeated attempts and updates were required only in two cases.

#### 2.2.4. Failure Occurrences Determination

Time instants of myocontrol failure occurrences were annotated exploiting two sources of information, the video recording of the full task and the button pressing of the subject. Whenever the user would realize that the prosthetic hand is not acting according to her intent, she should indicate this by a short press of the wireless button. By doing so we combined understanding of the participant about what was intended to happen with the details of video analysis. Detailed information on how exactly the two sources of information (user input and video annotations) were reconciled are provided in section 2.4.

#### 2.2.5. Instability Stress Factors

It is worth to highlight that the specific design of the experimental protocol was chosen in order to insert instability stress factors within the execution of the tasks. Indeed, sources of myocontrol instability during Activities of Daily Living (ADL) can be principally due to: mental effort and lack of concentration of the user during the execution of simultaneous duties (i.e., controlling the prosthesis with muscle contraction while walking, sitting, etc.); usage of small data sets for the training of the ML-based myocontrol algorithm; user tiredness; and variations of the body posture (with a particular consideration for the posture of the arm from where the sEMG data is acquired) (Fougner et al., 2011; Wolf et al., 2013; Khushaba et al., 2016). Therefore, the experimental task and protocol were designed in order to contain the following specific instability stress factors: increased possibility of misinterpretations of the ML myocontrol algorithm by training on multiple grasp types with only one repetition; training of the ML algorithm in a totally different body posture (in a sitting position with the elbow resting on the armrest) with respect to the one used during the task executions; variations of the body (and arm) posture in order to accomplish the task (standing, sitting, stretching in order to grasp and release in lower/higher locations, walk); and, finally, several task repetitions, which likely introduced non-negligible physical and mental efforts.

### 2.3. Feature Extraction

The feature types for the classifier were selected on the basis of the observation that an instability of the myocontrol is reflected as an oscillatory behavior on the prediction outputs of the related ML algorithm. Furthermore, according to the control mapping between the prediction signals and the motor currents for the control of the prosthetic hand (see section 2.1), in order to actually make a failure happen the prediction signal has to necessarily show an oscillation, for at least one of its four components. Therefore, this has to be reflected, to a certain extent, in the opening/closing motions of the fingers of the prosthetic hand (i.e., in a delayed and electromechanically smoothed form).

Accordingly, two different features were considered. One was the counting of the number of myocontrol prediction signal crossings of the threshold value of the prediction-to-motor currents mapping function (see section 2.1), or, for simplicity, *threshold crossings* (TC). It can be noted that such a feature, embedding the oscillating behavior aforementioned, does not directly depend on the prosthetic device, being computed just a step before the artificial hand subsystem is involved (see Figure 1). For the second feature types, the “digit status” feedback available from the i-LIMB (see section 2.1) was considered, which includes the behavior of the prosthetic hand in the feature space representation. In particular, the i-LIMB feedback was filtered to obtain a new signal, named “filtered digit status,” with only two possible values: “flexing” (replacing the closed/closing digit statuses) and “extending” (replacing the open/opening digit statuses). Specifically, the “extending” status was associated to the value 0, whereas the “flexing” status to the value 1. This way, the counting of the variations of the filtered digit status value—or simply *status changes* (SC)—was used as a feature. Both TC and SC features are computed by means of a running window; the operation performed over the window was simply the direct crossings and the status changes. Incidentally, note that the difference between TC and SC lies in the fact that the latter embeds information from the physical system of the prosthesis. Indeed, it is not possible in general to compute SC from TC: multiple consecutive prediction signal crossings of the threshold (incorporated by TC) will not be visible in SC if they are faster than the electromechanical response time of the artificial hand; additionally, when the prosthesis fingers are stalled due to external forces, crossings of the threshold will not be appreciable in SC, since SC varies only in relation to fingers physical motions.

Thereafter, from such features, we derived three different feature sets: one composed by the TC feature, another by the SC feature and, lastly, one by the combination of the two previous ones (denoted TC+SC). It follows that the related feature vectors are

$$\begin{array}{l} \;f_{TC}\;\in \;{\mathbb{N}}^{4},\\ \;f_{SC}\;\in \;{\mathbb{N}}^{4},\\ f_{TC+SC}\;\in \;{\mathbb{N}}^{8}, \end{array}$$

where *f*_*TC*_, *f*_*SC*_, and *f*_*TC*+*SC*_ are the TC, SC, and TC+SC (in the following also indicated as TCSC) feature vectors, respectively. In addition, three different window lengths were tested for the computation of the feature vectors, both without overlap and with an overlap of half the window length for each of them. In this way it was possible to evaluate the performance of the instability detection also in relation to the delays that the different feature extraction methods would introduce in an online implementation. Therefore, resuming for the sake of clarity, the whole evaluation was tested on three different feature *types*, computed with three different window length-window overlap couples, providing in total 18 feature *sets* for each of the subjects involved in the experiment.

### 2.4. Labeling and Classification

Firstly, for the sake of clarity, note that the automatic oracle was trained on a training set labeled thanks to the exploitation of the experimental task design and the video analysis, and thereafter was tested on new data (that was not part of the training set). In this testing, even if the experimental task phases were carefully designed, when a certain action of the prosthesis was performed (closing, opening or any other) the algorithm would not be able to predict a myocontrol failure, for example, with a simple congruency-check, because it doesn't know when and why such action was performed—a machine learning algorithm is required for this purpose. In particular, the labeling of the features, computed as described in the previous subsection, was performed by going through three steps: first, the beginning and end instants of time were obtained for each task; afterwards, a user's *intent ground truth* was determined in relation to the execution of the grasp or the rest action (see section 2.2); finally, the instants of time of failures were identified, allowing us to define the myocontrol behavior as stable or unstable.

**Figure 4:**
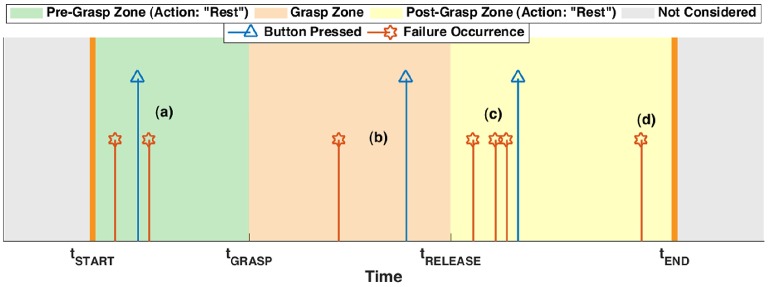
A qualitative example of the timing of a task, together with the user intent (that determines the different task zones, denoted with colored areas) and the failures. The latter were determined by video analysis (red stars), also exploiting the information reported by the subject pressing the wireless button (blue triangles). The letters (a–d) indicate the different instances of pressing the button in response to a failure, according to the list of section 2.4.

The starting and ending time of a task, namely *t*_*START*_ and *t*_*END*_, respectively, were input by the experimenter by pressing a key on the computer keyboard, while vocally indicating them to the subject. On the other hand, the user's intent ground truth is obtained by manually inspecting the video recording of each experiment (a timer was seen in the video, synchronized with the data recording, Figure 3) determining the times of the grasping and releasing of the mug, here indicated as *t*_*GRASP*_ and *t*_*RELEASE*_, respectively. In this way, considering that the subject is instructed—within every single task—to (i) rest from *t*_*START*_ until grasping the mug, (ii) grasp from *t*_*GRASP*_ until releasing it, and (iii) rest, again, from *t*_*RELEASE*_ till the end of the task *t*_*END*_, therefore it was possible to individuate the temporal evolution of the users' intent with a reasonable degree of reliability, and use it for the myocontrol performance labeling. A qualitative graph of the task and user intent timing is depicted in Figure 4. Thereafter, the instants of time in which a failure occurred where identified using the video analysis; more in detail, it was done by carefully looking at the behavior of the prosthetic hand and, once a behavior of the prosthetic hand in contrast with the user intent was identified, then the exact instant of time was determined by individuating the initial video frame of such behavior, and taking note of the time displayed in the timer present in the video. Exploiting the information given by the intent ground truth, the occurrence of motions of the prosthetic hand that were in disagreement with the user's intent was considered as “myocontrol failure” event, whereas every motion in accordance to the intent was considered as “myocontrol success.” It is worth to highlight that the determination of failure/success behaviors is based on *a posteriori* judgement based on video inspections which is, however, further supported by the information of the user's opinion provided by pressing the wireless button. In this way, the experiment scene was analyzed (e.g., slow-motion replay, frame by frame inspection, etc.) without neglecting the users feeling on the occurrence of a failure: this means that particular attention was devoted to identify failure time instants around the pressing of the wireless button by the user; however, at the same time, the entire video was carefully inspected for failures, and therefore, in case an unwanted behavior of the prosthesis occurred when not signaled by the user, or differently, there was no failure even if signaled by the user, the decision based on the mere video analysis was considered trustworthy. Indeed the online judgement of the user provided by pressing the wireless button cannot be considered absolutely reliable; in particular within the present study we were able to identify specific recurring unreliable behaviors (refer also to Figure 4):

- once a subject presses the button, she will not signal again for additional failures following the first instance (i.e., while and just after the button pressing);
- there is a “structural” delay between the occurrence of a myocontrol failure and the pressing of the button by the user—this is due to a cognitive process in identifying the failure, and the delay can be remarkable (of the order of seconds);
- during the delay mentioned in (2), if other failures occur then they are not reliably reported by the user;
- finally, even if the subject is asked to constantly focus on identifying unwanted behaviors of the prosthetic hand, some failures are not even noticed/perceived by the subject, and therefore they are simply not reported.

Therefore the video analysis allowed us to determine the existence and time instants of myocontrol failure occurrences bypassing the online-only judgement problems just listed above, which also formed the most relevant criticisms of our previous preliminar study on automated instability detection (Nowak et al., 2017b). Finally, note that the failures were discrete events (see Figure 4) because, in the study, the interest was related to the initial instant in which an unwanted behavior of the prosthetic hand manifested. The fact that a failure could persist after its initial occurrence were not considered as relevant, because the detection of a failure implies—in an online usage scenario—the stop of the action executed by the user and the performing of a model update.

At the end of the procedure just described, the outcome is a set of time instants of the myocontrol failures. This information was thereafter used to label the features extracted from the user-machine data flow (as described in section 2.3). In particular, since every feature vector corresponded to a certain data window, in accordance to the specific length and overlap used, if within the window a failure was present, then the label associated to such related feature vector was set to “failure” (associated to the value 1); in the opposite case, as “success” (associated to the value 0). The labeled features were used as input to a classifier in order to evaluate the automatic detection of an instability in the myocontrol.

A Support Vector Machine (SVM) classifier was used to perform the classification of the feature sets. The implementation was realized in MATLAB by means of the LIBSVM library (Chang and Lin, 2001). The procedure for the validation of the myocontrol failures detection was realized for each single subject by means of a nested cross-validation (CV) applied to the whole dataset, composed by the features extracted from the data of all the tasks executed during the experiment (i.e., 20 tasks, see section 2.2). Note that training dataset imbalances have been taken into consideration when training the classifier (especially in relation to the usage of different window lengths) using a class-weighted SVM (wSVM) (Akbani et al., 2004). The wSVM differs from the non-weighted variant because it uses two regularization parameters *C*_0_, for the class “failure” (value: 0), and *C*_1_, for the class “success” (value: 1), instead of using a single regularization parameter *C* (for details refer also to Veropoulos et al., 1999). Specifically, in order to tackle the skewness of class portions in the datasets, the regularization parameters of the wSVM were set as *C*_0_ = 1 and *C*_1_ = *n*_0_/*n*_1_, where *n*_0_ and *n*_1_ are the number of “success” and “failure” classes in the training dataset, respectively. Thus, in detail, the CV was composed by two nested loops. The inner loop consisted of a 10-fold CV, where a grid-search was conducted for the selection of the best wSVM classifier hyperparameter combination. The outer loop, a 10-fold CV as well, evaluates the performance of the wSVM model that won in the inner loop, tested on a separated external fold.

## 3. Results

### 3.1. Metrics and Statistical Analysis

First, an analysis of the myocontrol failure occurrences along the individual task executions is reported. Such analysis provides for an overview of the temporal distribution of the failures and, according to the structure of the experimental protocol, permits to retrieve preliminary considerations on the nature and distribution of the failures themselves.

Subsequently, a global evaluation of the classification performance was carried out considering all the six subjects involved in the experiment. Specifically, in order to provide an overview of the experimental results, we used the Receiver Operating Characteristics (ROC) (Fawcett, 2006), and computed the balanced error rates (BER) over the different combinations of window lengths and overlaps for the extraction of the features. Furthermore, the data gathered from the subjects was also evaluated statistically. Here, we performed a three-way ANOVA with the factors *feature type, window length*, and *window overlap*, in order to determine the set of parameters which provides the best performances. Finally, due to the modest number of subjects involved in the present “first step” study on automatic myocontrol failure detection, we conducted a statistical power analysis related to our experimental design.

### 3.2. Per-Subject Failure Occurrence Analysis

In this section a per-subject analysis of the failure occurrences is provided, intended to launch the global analysis on the classification performance of the following subsection, which otherwise could result incomplete since decoupled from a view of the number and temporal distribution of the myocontrol failures. In the following, the subjects involved in the experiments will be indicated as S1, S2, S3, S4, and S5 for the able-bodied ones, and SA for the amputee.

**Figure 5:**
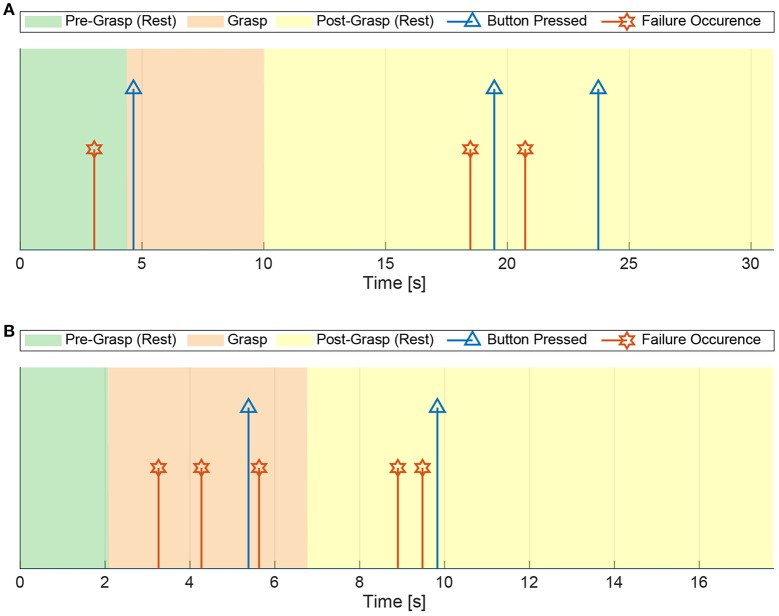
Real-case example of the temporal plot of the myocontrol failures and the wireless button pressings within a task execution, for the subjects S4 and SA. The failure occurrences are determined by the *a posteriori* video analysis of the experiments. **(A)** Eighth task of the subject S4. **(B)** Twelfth task of the subject SA.

Fist of all, an example of the temporal plot of the myocontrol failures along with the wireless button pressings is provided in Figure 5, in particular for one task of the subjects S4 and SA. Such graphs are the equivalent realistic case of the qualitative graph of Figure 4. For the sake of compactness, the temporal plots of the failures for all the tasks and subjects are not reported, instead the number and temporal distribution of the failures were analyzed in an aggregate form along the different task executions, considering each subject individually.

**Figure 6:**
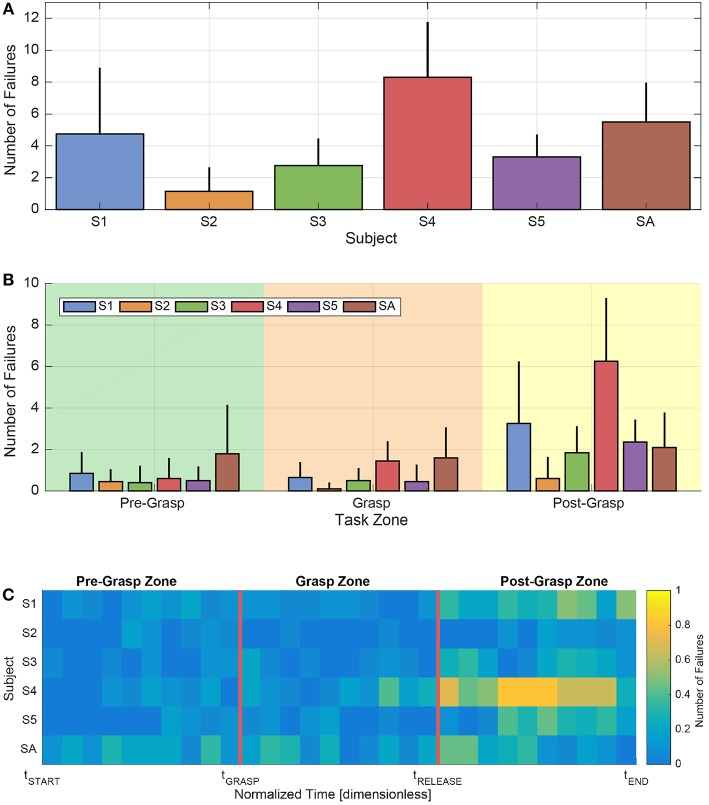
Analysis of the number and temporal distribution of the myocontrol failures, also in light of the different zones of the task executions, according to the experimental protocol and considering each subject singularly. **(A)** Mean number of failures along tasks for each subject. Error bars represent standard deviation. **(B)** Mean number of failures in the different zones of the tasks for each subject. Error bars represent standard deviation. **(C)** Map of the temporal behavior of the mean number of failures for each subject, computed on time portions normalized with respect to the different zone durations of the task executions.

Figure 6A shows the mean number of failures per task related to each subject. In particular, we can see that the highest mean number of failures was given by the subject S4, while the highest standard deviation can be observed in relation to the subject S1. From this preliminary analysis (together with the example provided in Figure 5), it is already possible to notice that the general behavior of the myocontrol failures for the amputee subject SA was comparable with the ones presented by the able-bodied subjects. This allows a global analysis of the failure detection performance including all subjects (see next subsection). Then, for a finer analysis, in Figure 6B the mean number of failures per subject is reported for the different zones of the task executions, that are: *Pre-Grasp* (between *t*_*START*_ and *t*_*GRASP*_), *Grasp* (between *t*_*GRASP*_ and *t*_*RELEASE*_) and *Post-Grasp* (between *t*_*RELEASE*_ and *t*_*END*_)—see section 2.4. From such figure it is possible to observe that the mean number of failures appreciably increased for all the subjects during the last part of the task execution (i.e., the Post-Grasp zone). This could be due to the combination of two aspects: the first was the lack of concentration due to the negative effect of mental and/or physical efforts in executing the task; the second was related to the duration of the Post-Grasp zone, which was found to be longer than the other zones for all subjects.

In order to further analyse the possible presence of patterns of failure occurrences within the task zones themselves, in Figure 6C a map of the temporal trend of the mean number of failures is provided. Specifically, such a map was computed according to a task-zones-wise normalized time, in order to make the various task durations uniform. Looking at the figure, first of all it is possible to better see that, on average, the myocontrol failures were mostly concentrated in the Post-Grasp zone of the task execution, and particularly in its central part. Furthermore, it is visible that at the beginning of the task, specifically in the first part of the Pre-Grasp zone, the mean number of failures was particularly limited (in several zones, zero). Differently, an increasing of failures was present as the normalized *t*_*GRASP*_ time was approached (i.e., the *reaching to grasp* phase), in which the motions of the arm and of the whole body of the subjects were particularly stressed (see frame 2 in Figure 3). Lastly, we can highlight that, in the Grasp zone, the mean number of failures was comparable with the values observable in the Pre-Grasp zone; in particular, those subjects that presented a higher average number of failures while holding the object, showed such behavior more in proximity of the starting and ending parts of the object grasp action. We are able to observe a certain degree of consistency among the Pre-Grasp and the Grasp zones, characterized by a clearly lower average number of failures with respect to the Post-Grasp zone.

### 3.3. Global Evaluation

#### 3.3.1. Classification Performance

**Figure 7:**
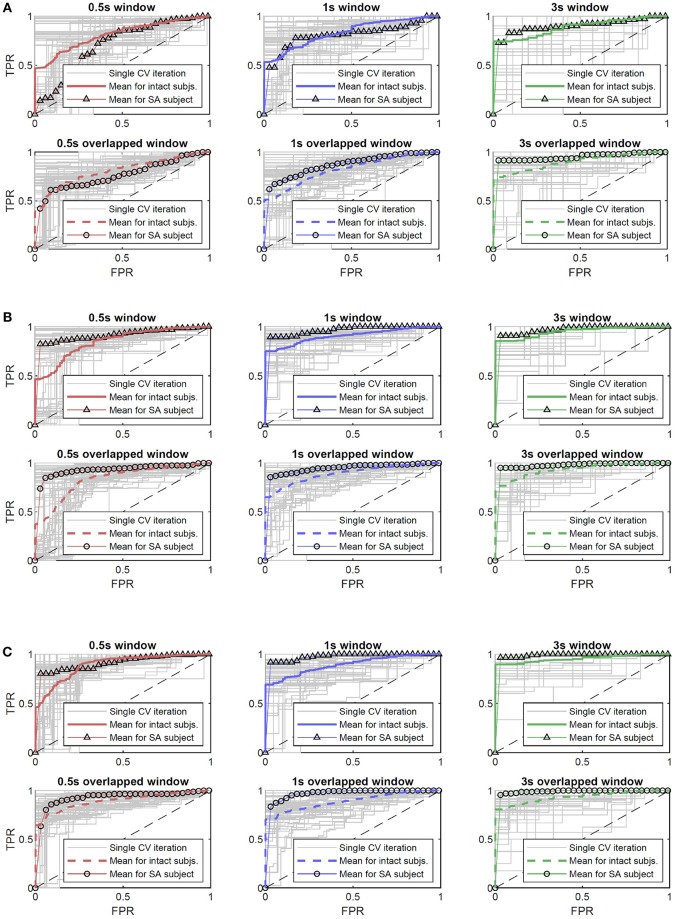
The mean ROC curves for the amputee and intact subjects, with respect to the selected type of feature, and reported for the different combinations of window length and overlap. In gray, the ROC curves obtained for each subject during the outer loop of the nested CV; red, blue, and green standard lines indicate the ROC curves related to intact subjects for the TC, TCSC, SC features, respectively, and the triangle-marked lines indicate the amputee. Dashed and circle-marked lines indicate the half window overlap for intact subjects and amputee, respectively. **(A)** ROC curves for the feature Threshold Crossing (indicated by TC). **(B)** ROC curves for the feature Threshold Crossing plus Status Change counting (indicated by TCSC). **(C)** ROC curves for the feature Status Change counting (indicated by SC).

The ROC curves plot the true positive rate vs. the false positive rate obtained by modifying the decision criterion of the wSVM class membership probabilities output, and were computed for every feature set, for each subject, in accordance with the convention illustrated in the following. A “myocontrol success” (or “success,” S) was considered the *positive condition* (P), whereas a “myocontrol failure” (or “failure,” F) was considered as the *negative condition* (N). In accordance with this, it was possible to define the relative *true positive* (TP), *false positive* (FP), *false negative* (FN) and *true negative* (TN) occurrences, that can be arranged in a confusion matrix *M*_*conf*_ of the form

$$\begin{array}{l} M_{conf}=\left[\begin{array}{cc} N_{TP} & N_{FP}\\ N_{FN} & N_{TN} \end{array}\right], \end{array}$$

where *N*_*TP*_, *N*_*FP*_, *N*_*FN*_, *N*_*TN*_ are the total number of TP, FP, FN, and TN occurrences based on the wSVM classifier prediction outputs, obtained during a single validation of the outer loop 10-fold CV (see Labeling and Classification subsection) for a given feature set. In this relation, the matrix of occurrence rates *M*_*rate*_ is given for each couple of subject and feature set as

(1) $$\begin{array}{l} M_{rate}=\left[\begin{array}{cc} TPR & FPR\\ FNR & TNR \end{array}\right]\\ \;=\;\left[\begin{array}{cc} \frac{N_{TP}}{N_{TP}+N_{FN}} & \frac{N_{FP}}{N_{FP}+N_{TN}}\\ \frac{N_{FN}}{N_{TP}+N_{FN}} & \frac{N_{TN}}{N_{FP}+N_{TN}} \end{array}\right]\;, \end{array}$$

where *TPR*, *FPR*, *FNR*, and *TNR* are the TP, FP, FN, and TN rates, respectively. Figure 7 therefore reports the mean ROC curve—over the five intact subjects and for the amputee—of the ROC curves obtained from the *TPR* and *FPR* of the matrices computed as in (1). Furthermore, Table 1 reports the Area Under Curve (AUC) of the mean ROCs of Figure 7: indeed the AUC provides an aggregate measure of the quality of the classifier model's predictions across all possible classification thresholds applied to the decision values; for further details about ROC and wSVM decision values see Fawcett (2006) and Chang and Lin (2001).

**Table 1:** The AUC values with respect to the ROC curves illustrated in Figure 7, reported for the different type of feature and combinations of window length/overlap, for the intact subjects (S1–5) and the amputee (SA).

		**AUC**					
**Feature type**	**Window overlap**	**0.5 s window**		**1 s window**		**3 s window**	
		**S1–5**	**SA**	**S1–5**	**SA**	**S1–5**	**SA**
**TC**	No overlap	0.7955	0.7172	0.8286	0.8108	0.8895	0.9031
	Overlap	0.7986	0.7656	0.8274	0.8731	0.9075	0.953
**TCSC**	No overlap	0.86	0.9196	0.9159	0.9662	0.9526	0.9722
	Overlap	0.8514	0.9345	0.9017	0.9523	0.942	0.9815
**SC**	No overlap	0.897	0.9195	0.9075	0.9808	**0.9606**	**0.9927**
	Overlap	0.9003	0.9304	0.9044	0.9755	0.9425	0.9915

*Numbers in bold highlight the higher AUC values for S1–5 and SA*.

In relation to these figure and table, let us firstly observe the results given by the TC feature type. Window lengths of 0.5 s report for lower AUC values, under the threshold of 0.8 (both for intact subjects and amputee), highlighting the presence of a relatively consistent number of missed or wrong failure detections. Instead, with window lengths of 1 s and 3 s we can see AUC values grater than 0.8, with the highest one equal to 0.9075 and 0.953 for the intact subjects and the amputee, respectively, for a 3 s overlapped window. Differently, looking now at Figure 7 and Table 1 for the TCSC and SC feature types, it is possible to observe better classification performance, i.e., AUC values always greater than 0.8, and greater than 0.9 if only the window lengths of 1 s and 3 s are considered. In particular, for the 3*s* window length, we can see that the minimum AUC value is equal to 0.942. Here, we can observe the preferable classifier model's performance for the case of a 3 s non-overlapped window: an AUC of 0.9606 for the intact subjects and 0.9927 for the amputee. Therefore, better classification performance are reported for the same combination of selected features for both intact subjects and amputee, also showing similar trend of performance along all the features/windowings. The overall best score was reported by the amputed subjects with an AUC of 0.9927.

**Figure 8:**
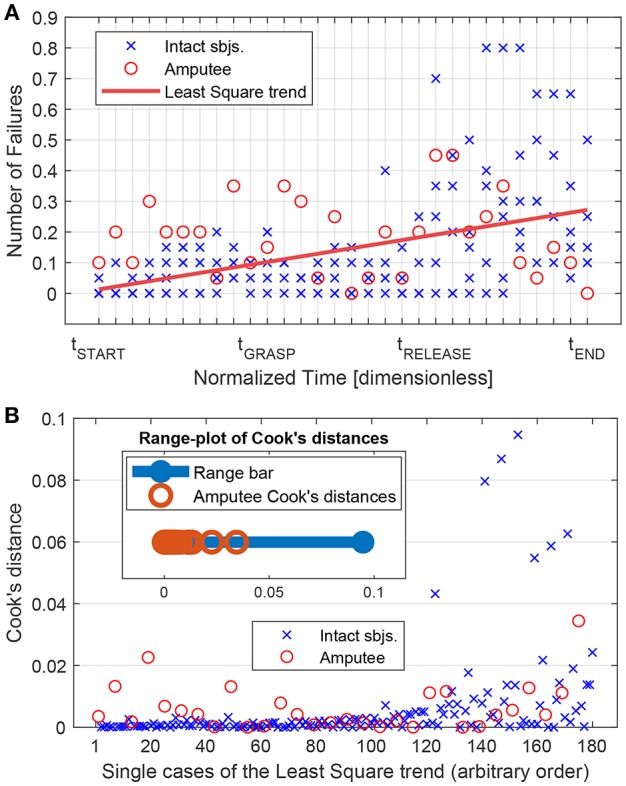
Influence of the amputee subject on the linear trend of the number of failures along the different phases of the experimental task (pre-grasp, grasp, post-grasp zones—see section 3.2). **(A)** All subjects Least Square linear trend (red line) of the mean number of failure computed on time portions normalized with the respect to the different zone duration (see Figure 6C and section 3.2). Points regarding the amputee subjects are reported with red circles, wheares blue crosses represent the points related to intact subjects. **(B)** Cook's distances of each single point (or *case*) related to the Lest Square trend of **(A)**. In the top-left part, it is present an aggregated range-plot of the Cook's distances, highlighting the location of the amputee-related values with red circles within the whole interval of distance values.

In order to further evaluate the failure detection performance and statistically assess their distribution among the subjects, the BER was computed for the different feature types and window length/overlap combinations. The BER is given as

$$\begin{array}{l} BER=\frac{100}{2}\left(\frac{N_{FN}}{N_{TP}+N_{FN}}+\frac{N_{FP}}{N_{FP}+N_{TN}}\right), \end{array}$$

where the multiplication by 100 is present to obtain a percentage value. The goal of the statistical analysis was to use the BER outcome metrics to compare different features and windowing characteristics. In order to do that, we first carried out a comparison between the intact subjects and the amputee. This has been done in two directions: (i) looking at the temporal distribution of the failures in the tasks and (ii) looking at the BER outcome. In relation to the distribution of the failures along the task, the temporal trend of the failures—as already illustrated in Figure 6C—was considered. Indeed, in Figure 8A such temporal trend is illustrated among all the subjects in a least square sense, highlighting the cases related to the amputee (red circles) and the cases related to the intact subjects (blue crosses). Then, the influence of each single case contributing to the least square trend was assessed computing the related Cook's distances—a commonly used estimate of the influence of data points in least-squares regression analyses (Cook, 2000)—which values are reported in Figure 8B. In particular, within this latter figure, it is also depicted an aggregated range-plot that highlights how the Cook's distances related to the amputee (marked with red circles in the range-plot) are not exceeding the middle point of the range of all distances, therefore meaning that the influence of the amputee on the temporal trend of the failure was limited with respect to the entire group of subjects. On the other hand, from the point of view of the BER metrics, the presence of a significant difference between amputee and intact subjects was verified, computing the BERs for all the combination of feature and windows, grouping then the data as “amputee” and “intact”. To do this, firstly the normality of these data was assessed using the Shapiro-Wilk test, which reported that the distribution of the group “amputee” significantly differed from a normal distribution (*p* = 0.042; significance level set to 0.05). Consequently, we performed the non-parametric Wilcoxon rank-sum test, which results reported that the BERs of the “amputee” group did not differ significantly from the “intact” group, *W* = 1021, *p* = 0.0826. Therefore, given the limited influence of the amputee on the overall temporal trend of failures during the tasks and the non-significant difference between the BERs of the intact subjects and the amputee, for the main statistical analysis related to the comparison of the different features and windowing combinations we pooled the data of the intact subjects and the amputee together for a global evaluation of the classification performance.

**Figure 9:**
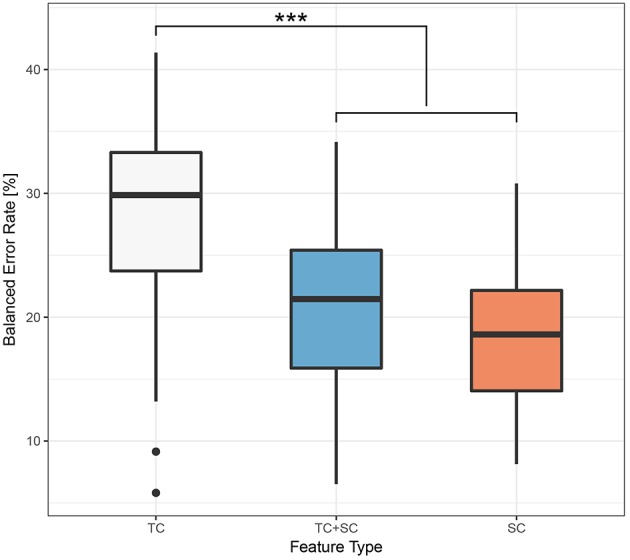
Boxplot of the BER grouped by *feature type*. The symbol “^***^” denotes *p* < 0.001, according to the Tukey-Test of section 3.3.

Therefore, in this relation, we investigated three different factors with the BER outcome, namely *feature type* (TC, TC+SC or SC), *window size* (0.5s, 1.0s or 3.0s) and *window overlap* (no overlap or half overlap). Taking these factors into account we performed a three-way ANOVA using the statistical tools provided in *R* (R Core Team, 2013). Normality of data distribution was verified using the Shapiro-Wilk test, and a Levene's test to check the homogeneity of variance was performed; both tests revealed that the assumptions of the ANOVA were not violated. Statistical significance was set to *p* < 0.05. We obtained the following results: for the factor *feature type F*(2, 90) = 17.493, *p* < 10^−3^, for the factor *window size F*(2, 90) = 2.809, *p* = 0.066 and for *window overlap F*(1, 90) = 1.866, *p* = 0.175. Since *window size, window overlap* and all interaction terms as well have no significant influence, as usual in the case of nonsignificant factors we pooled together the related data, reducing the model to the only factor *feature type*, and performed a one-way ANOVA. The results are *F*(2, 105) = 18.09, *p* < 10^−3^. A boxplot of the different groups can be found in Figure 9. The one-way ANOVA was then followed up by a Tukey-test to perform pairwise comparisons. It followed that both SC and TC+SC perform significantly better than TC with *p* < 10^−3^ in both cases. However, the difference between SC and TC+SC is not significant with *p* = 0.569. The Tukey-test therefore revealed that better classification performance are obtained with the SC and TC+SC feature types.

**Figure 10:**
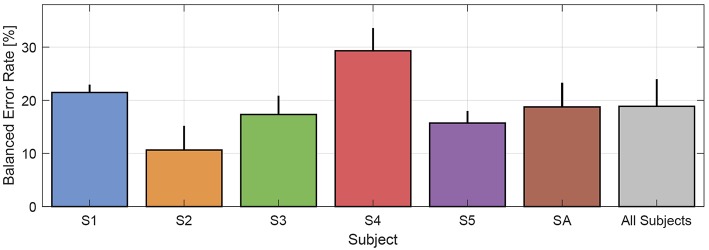
Mean BER per each subject over the single BERs obtained with both TC+SC and SC features. The resultant mean BER over all subjects is equal to 18.86%.

Finally, we report in Figure 10 the mean BER per each subject over the single BERs obtained with both the TC+SC and SC feature types (since we have seen that there is no statistically significant difference between TC+SC and SC). Looking at the figure, the resulting mean BER over all the subjects is equal to 18.86%. This classification performance provides positive outcomes on the actual possibility to automatically detect instability with the mid-term prospect of improving myocontrol reliability.

#### 3.3.2. Statistical Power and Results Generalizability

In order to analyse the power of our experimental design in detecting statistical effects, we conducted a *post hoc power analysis* with the program G^*^Power (Faul et al., 2009; for a full description see Erdfelder et al., 1996), with relation to key main effects taken into account with the ANOVA of the previous subsection. Let us first consider the significantly better performance of the features SC and TCSC with respect to TC resulting from the one-way ANOVA. We have a total sample size of 108 and a number of groups equal to 3. The related observed effect size calculated on the basis of the sum of squares was equal to *f* = 0.587 [that is a large effect size (Cohen, 1988)]; the power (1 − β) to detect an effect of this size was determined to be 0.9889 (for a significance set at α = 0.001), which was clearly greater than the (conventionally) recommended threshold of 0.8 (Cohen, 1988). On the other hand, considering now the effect of the factors statistically analyzed by the three-way ANOVA in the previous subsection (total sample size of 108; number of groups equal to 18), we calculated on the basis of the sum of squares that the effect size of the factor *feature type* was 0.6235, and the related power of the experiment was 0.9955 (for a significance set at α = 0.001), clearly greater than the conventional threshold of 0.8. Differently, the effect size of the factor *window length* was calculated to be equal to 0.2496 with a related power of the experiment (1 − β) = 0.1458 (for a significance at α = 0.001) and (1 − β) = 0.6215 (for a significance at α = 0.05). The effect size of the factor *window overlap* resulted to be equal to 0.1436 with a related power of the experiment (1 − β) = 0.0327 (for a significance at α = 0.001) and (1 − β) = 0.3147 (for a significance at α = 0.05). Therefore, we cannot completely rule out that there was an effect of the factors *window length* and *window overlap* as resulting by the three-way ANOVA, because it exists the possibility that the limited statistical power have played a role in limiting the significance of such specific factors. Indeed, to obtain a power of at least (1 − β) = 0.8, a total sample size of 834 (with significance at α = 0.001) or 383 (with significance at α = 0.05) would be needed for the *window overlap* factor, and a total sample size of 323 (with significance at α = 0.001) or 158 (with significance at α = 0.05) would be required for the *window length* factor. However, what we can say is that the experiment had enough statistical power to detect a significant better classification performance given by the usage of the SC and TCSC feature.

In addition, there are also some considerations that must be outlined about possible limits in the generalizability of the presented results. First of all, as already mentioned in section 3.1, notwithstanding the importance that we think this first study on Interactive Myocontrol has, it is necessary to take into account that the number of six subjects involved in the experiment was relatively modest. In this regard, pooling together the intact subjects and the amputee for the ANOVA reported in section 3.3.1 has to be handled with care, even if motivated by an analysis of the influence of the amputed subject on the overall temporal distribution of the failures and, more directly, on a Wilcoxon rank sum test (see section 3.3.1). Indeed, there exists the risk that the number of intact subjects *N*_*I*_ = 5 and the number of amputees *N*_*A*_ = 1 could limit the validity of such results to only the specific subjects involved in this study, therefore affecting its generalizability. In fact, it is necessary to take into account that in real daily life applications amputees could show remarkable levels of heterogeneity with respect to able-bodied subjects and to other amputees. However, if such differences affect also the sphere of automatic failure detection for Interactive Myocontrol still needs to be verified, and further studies have to be carried out also in this direction, as outlined in the following section 4. In this study, for a separated analysis and comparison of the failure detection performance between the intact subjects and the amputee, it is possible to refer to Figure 7 and Table 1 and related explanations in section 3.3.1.

## 4. Discussion and Conclusions

### 4.1. Interactive Myocontrol and Co-adaptation

The study presented in this article took its inspiration from the *interactive myocontrol* paradigm as a way to face the problem of instability in myocontrol, so far unresolved. In interactive myocontrol, the s/p control scheme can be updated with new information, once necessary in the face of a *myocontrol failure* occurrence—as a particularization of the incremental learning concept. Within the whole framework of human-machine interaction in prosthesis control, it is desirable to have such incremental updates in an autonomous and real time fashion, and therefore we introduced an *automatic oracle* agent as a counterpart of a “human oracle,” whose presence we want to avoid because it represents a source of bias of the whole paradigm. The role of the automatic oracle is to detect when a myocontrol failure occurs, defined as a behavior of the prosthetic device that does not coincide with the user intent. Indeed, please note that the goal of the myocontrol model was exactly to translate the muscle output signals into the user intent: therefore, if during the prosthesis usage the muscle output became flawed for any reason, then the model should understand that such specific output was still related to a certain intent. It follows that failure detection is needed to let the model gather more data and understand that new muscle signals have to be considered in order to continue understanding the right intent of the user. Finally, theoretically the action of the automatic oracle should be as much transparent and imperceptible as possible for the user, i.e., the user should not notice its presence even during the update of the s/p myocontrol.

On the other hand, we are aware that total transparency is very difficult to achieve—if not impossible—and indeed, interactive myocontrol also aims to *co-adapt* with the user. Picture putting the automatic oracle together with the subject's judgement: once a failure is detected, the system would ask to the user to repeat the action while she is communicating her intent, in order to let the oracle gather new data in view of the incremental learning. Thereafter the myocontroller model update will occur automatically with basically no additional burden to the user; in other specific cases the subject could voluntarily “teach the prosthesis” for new data to preventively improve myocontrol stability, or even to learn completely novel actions.

In the light of these concepts, the authors firmly think that the topic of automated instability detection is quintessential to fully exploit interactive myocontrol. We are therefore confident that the work here presented, focusing on the possibility of classifying myocontrol instability, represents an essential preliminary step toward a truly interactive prosthetic myocontrol.

### 4.2. Informational Set for Automated Failure Detection

Figure 9 shows that statistically significantly better classification performances were obtained using the TC+SC and SC feature types, whereas there was no significant difference between TC+SC and SC (refer to the ANOVA results in the section 3.3). This result tells that the usage of the information coming from the prosthetic device significantly improved the classification performance, highlighting the importance of the terminal part of the user-device interface chain, i.e., the informational zone that is the outcome of the influences of all the previous steps (the *human-machine interface informational zone*—refer to Figure 1). In light of the obtained results, this can be likely due to the fact that part of the information embedded in the TC feature was not corresponding to any behavior visible to a human eye (for details see section 2.3)—even in a meticulous video analysis—and therefore was simply not corresponding to a myocontrol failure, determining poorer classification performance with the TC feature only, in accordance to the importance of the information given directly by the prosthetic device.

Additionally, the dimension of the informational set needed for the automatic oracle is worth to be discussed. In principle the inclusion of all possible data from the HMI environment is absolutely welcome (Figure 1): just think about an automatic oracle that can get as much knowledge as possible from the prosthetic system in order to better understand myocontrol failure occurrences and be potentially very reactive to unknown situation—the more information available, the better the performance. Nevertheless, the experimental results presented in section 3.3 shows that a failure classification performance with mean BER of 18.86% (refer to Figure 10) can be obtained with a very small subset of the available HMI information, i.e., only using the prosthetic hand feedback about the extending/flexing status of the fingers (SC feature). This specific aspect deserves a careful consideration, because it shows the possibility to improve the reliability of myocontrol with a very limited set of information and, therefore, with few hardware requirements (e.g., sensors). This means lower costs without negatively affect the user's degree of acceptance of the prosthetic system (which is strongly influenced by weight, heat production, hardware faults, etc.).

### 4.3. Can the Automated Detection Go Online?

One of the fundamental aspects to detect myocontrol failures online (and possibly update the myocontroller model on-the-fly) is the window length used to extract the features for the classifier, because of the delay that it consequently introduces. The results show that the classification performance were independent of the selected window length and related overlap (refer to Figures 7, 9) and that there was a statistically significant improvement in the performance as the TC+SC or SC feature types are used (refer to section 3.3, Tukey-Test results). This means that acceptable classification performance can be obtained introducing only a relatively contained delay of 0.25 s on top of the 0.5 s window length. Indeed, in order to enhance the immediacy of the failure detection, it is preferable to have shorter time windows, approaching lengths of 100—200 ms (that are close to the delay perception limit for humans). On the other hand, notice that a fast response to a failure occurrence is not a founding specification for the interactive myocontrol framework. Indeed, before a failure is detected, some data has to necessarily be logged in order to allow the incremental update of the myocontroller model (a query to the user to know which would have been the correct action could be necessary, if not only the “power grasp” is supposed to be used for grasping). Therefore, in this view, a certain delay would be unavoidable—even desirable: the data could continuously be logged by means of the window for the features extraction itself, and then automatically provided for the update when a failure is detected. Even a delay of few seconds could turn into a desirable feature. For these reasons, we think that the possibility to classify myocontrol instability emerging from the results nicely fits the interactive myocontrol framework, providing remarkable prospectives in the right direction for further studies toward an online implementation of the application.

Another important aspect to make the automatic failure detection go online is the determination of the user intent “ground truth” necessary to label the training data for the automatic oracle. In the present work, the definition and timing of the user intent was possible by the combination of *a priori* (design of the experimental task) and *a posteriori* (video analysis) actions, which are difficult to be applicable for real world applications. However, note that our study focused on verifying the actual possibility to build a classifier able to detect myocontrol instabilities. Being this work a novel approach and direction of research in this field, for the moment we didn't want to respond to the question “how to determine user intent ground truths in real world applications for the automatic oracle trainings,” instead our experimental goal was to find “how to have a reliable user intent ground truth for the data labeling of the automatic oracle, in order to verify if myocontrol failure detection is actually possible.” Aspects relating the determination of reliable user intent ground truths in real world applications will be object of future studies.

### 4.4. A Wider Perspective: Myocontrol and Radical Constructivism

In Nowak et al. (2018) we speculated that myocontrol, as instantiated by ML, could benefit from a *Radical Constructivist* (RC, standing also for Radical Constructivism) approach. RC is a branch of constructivist psychology positing that learning, as a generic process in humans as well as in machines (i.e., the *agents*), is an attempt to optimally organize the agent's perceptive field, i.e., its own sensory and experiential inner world, according to some specific fitting criterion (von Glasersfeld, 1983, 1995). Once it becomes incremental and interactive (Gijsberts et al., 2014; Strazzulla et al., 2017), myocontrol fits quite well in this picture: it is the attempt of a ML algorithm to fit as best as possible the bio-signal patterns received from a disabled human subject. One of the crucial aspects of RC-framed myocontrol is then the necessity of having a reliable “oracle” allowing qualitative feedback be sent back to itself: whenever something goes wrong (i.e., a myocontrol failure), based on this knowledge the ML model must take action to correct its own perceptive landscape; as well, a good fit can be reinforced.

So far, the feedback oracle has been the user herself. Thanks to interactive learning, she has been able to ask for further data gathering and model updating whenever required. In RC terms, we view the quest for an *automated* oracle—that we have introduced in this paper—as an attempt to enlarge the perceptive universe of the machine (Nowak et al., 2018).

### Conclusions

In this article we presented the results of a study for the improvement of the myocontrol of prosthetic hands with respect to the well-known issue of reliability. We outlined the concept of “automatic oracle,” i.e., a supervising agent that is able to classify when a myocontrol failure occurs, and to carry out an incremental learning paradigm to deal with myocontrol instability. Relying on such general framework, in this work we focussed on the possibility of classifying the myocontrol instability testing a set of features extracted from the user-device interface's informational chain. To this purpose, we engaged six expert myocontrol users (five able-bodied persons and one trans-radial amputee) in a simplified and carefully designed experiment. It consisted of 20 grasping tasks for each subject, in which we were able to identify the exact timing of myocontrol failure occurrences thanks to the information provided by the users and to an offline analysis of the experiment video recordings, as detailed in section 2.3. In this way, exploiting reasonable assumptions on the subjects' intent based on the structure of our experimental protocol, it was possible to label the features as “myocontrol success” or “myocontrol failure,” in order to train and test a wSVM classifier for the automated detection of myocontrol instability.

We are confident that the work presented in this article represents a further step toward a truly interactive prosthetic myocontrol, in which the overall HMI system will be capable of online detecting myocontrol instability, allowing for proper model updates and virtuous user-device interactions where necessary. About forthcoming future work, the focus will firstly go on improving the failure classification accuracy by investigating the usage of different and/or more advanced feature typologies. We also want to work on a generalization of the failure definition and detection, for example considering the teleoperation of ideal and “fully programmable” artificial hands, as it is possible in presence of virtual reality scenarios. Thereafter, future efforts will be devoted to the implementation of the real online application. Such kind of studies are fascinating also in relation to the possible applications in non-prosthetic or -rehabilitation systems, i.e., in general teleoperation in the field of human-robot interaction.

## Ethics Statement

This study was carried out in accordance with the recommendations of the local ethics committee (Ethical Committee of DLR) with written informed consent from all subjects. All subjects gave written informed consent in accordance with the Declaration of Helsinki. The protocol was approved by the Ethical Committee of DLR.

## Author Contributions

RM, MN, CM, and CC planned the study and the experiments. RM and MN prepared and conducted the experiments. RM postprocessed the experimental data and produced the results. RM, MN, and CC analyzed the results and RM created the figures. RM, MN, and CC wrote the manuscript. All authors reviewed the manuscript for important intellectual content and approved the submitted version.

### Conflict of Interest Statement

The authors declare that the research was conducted in the absence of any commercial or financial relationships that could be construed as a potential conflict of interest.

## Footnotes

^1^The winners are Robert Radocy of TRS Prosthetics (TRS-Prosthetics, 2016) and the Softhand Team (Godfrey et al., 2017).
